# Delivery Systems of Retinoprotective Proteins in the Retina

**DOI:** 10.3390/ijms22105344

**Published:** 2021-05-19

**Authors:** Ivan T. Rebustini, Alexandra Bernardo-Colón, Alejandra Isasi Nalvarte, S. Patricia Becerra

**Affiliations:** Section of Protein Structure and Function, LRCMB-NEI-NIH, Bethesda, MD 20892-0608, USA; alexandra.bernardocolon@nih.gov (A.B.-C.); alejandra.isasinalvarte@nih.gov (A.I.N.)

**Keywords:** retinoprotective, peptide, retina, photoreceptors, pigmented epithelium-derived factor, delivery systems

## Abstract

Retinoprotective proteins play important roles for retinal tissue integrity. They can directly affect the function and the survival of photoreceptors, and/or indirectly target the retinal pigment epithelium (RPE) and endothelial cells that support these tissues. Retinoprotective proteins are used in basic, translational and in clinical studies to prevent and treat human retinal degenerative disorders. In this review, we provide an overview of proteins that protect the retina and focus on pigment epithelium-derived factor (PEDF), and its effects on photoreceptors, RPE cells, and endothelial cells. We also discuss delivery systems such as pharmacologic and genetic administration of proteins to achieve photoreceptor survival and retinal tissue integrity.

## 1. Introduction

The neural retina, composed of layers of photoreceptors, horizontal cells, bipolar cells, Müller cells, amacrine cells and retinal ganglion cells, converts light stimulation into chemical signals sent through the optic nerve into the brain [1]. The crucial process of visual phototransduction occurs in the photoreceptors, which are highly specialized and terminally differentiated cells, and involves light-sensing opsins. In most species of vertebrates, adult photoreceptors do not proliferate and finish their differentiation at early postnatal stages [2]. Damage of photoreceptors and their supportive tissues, such as the retinal pigment epithelial (RPE) cells, causes multiple forms of retinal pathogenesis [3]. Retinoprotective proteins can prevent cell death of photoreceptors and RPE cells while maintaining cellular functions [4]. Another important role of retinoprotective proteins includes inhibiting angiogenesis that dysregulates blood vessels in the retina and generates pathological phenotypes observed in retinal degeneration [5].

In this review, we offer an overview of retinoprotective proteins that target retinopathies including their mechanisms of action (Section 2), and focus on pigment epithelium derived factor (PEDF, Section 3). We also describe systems of local delivery of PEDF into the retina (Section 4): (1) pharmacologic administration of PEDF (recombinantly produced proteins, extracted and purified from bacterial and mammalian cells) or their derived peptides (chemically synthesized peptide fragments from larger, modular proteins containing biologically active regions); (2) genetic administration of the *Serpinf1* gene achieved by infections or transfections of expression vectors encoding for PEDF in the retinal or in the RPE tissues in vivo; and (3) Cell-based systems in which PEDF-expressing cells are encapsulated in vitro and transplanted into the retina in vivo for cell therapies are also discussed.

## 2. Overview of Retinoprotective Proteins

Retinoprotective proteins function either via autocrine signaling (when expressed and secreted by the same cells that they target), paracrine signaling (when expressed by supporting tissues such as the RPE to target photoreceptors) or endocrine signaling (when delivered from distal tissues to the photoreceptors by the choroidal and the inner retinal vasculature) [4]. Representative examples of retinoprotective proteins are briefly discussed and summarized in the Table 1.

### 2.1. Autocrine Retinoprotective Proteins

Naturally occurring proteins that act in an autocrine fashion during retinal development can be administered postnatally as retinoprotective agents. Among the most studied of these proteins include Insulin-like Growth Factor 1 (IGF1, [6]), Fibroblast Growth Factor 2 (FGF2, [7]), Ciliary Neurotrophic Factor (CNTF, [8]), Brain-Derived Neurotrophic Factor (BDNF, [9]), Nerve Growth Factor (NGF, [10]) and Vascular Endothelial Growth Factor (VEGF, [11]). The re-induction of IGF1 [18,19] and FGF2, which is retinoprotective to photoreceptors by regulating Ca^2+^ influx in a model of retinitis pigmentosa [7] are examples of retinoprotective proteins that are developmentally regulated in the retina, and can contribute to adult photoreceptor homeostasis when pharmacologically or genetically delivered in the retina. Studies with *cntf* KO mouse reveal that CNTF can protect cones and rods by suppressing excessive formation of visual pigments [8]. Recombinant human NGF promotes photoreceptor survival in a rat model of retinitis pigmentosa [10]. An example of the application of autocrine retinoprotective proteins for retinal protection is the use of 7,8-dihydroxyflavone hydrate (7,8-DHF), which is a drug that is a mimetic to BDNF [9], in combination with histone deacetylase inhibitors (HDACi) currently in clinical trial for the treatment of inherited retinal degeneration. The individual use or the combination of the BDNF mimetic and HDACi are capable to restore photoreceptors visual function in a zebrafish *dye* ^ucd6^ blindness model [9]. An overview of representative autocrine retinoprotective proteins is summarized in the Table 1.

### 2.2. Paracrine Retinoprotective Proteins

Representative examples of paracrine retinoprotective proteins include the pituitary adenylate cyclase-activating polypeptide (PACAP) and vasoactive intestinal peptide (VIP, [12]), somatostatin [20], and Vascular Endothelial Growth Factor (VEGF, [11]). VIP is expressed in a population of amacrine cells [13], whereas PACAP and its receptors (PAC1, VPAC1, and VPAC2) occur in all retinal layers except the photoreceptors [14]. Topically administered PACAP and VIP derivatives in the form of eye drops attenuate ischemic retinal degeneration [21]. Of note, IGF1, previously mentioned above as possessing an autocrine signaling mechanism of action, can also act in a paracrine manner (Table 1), as it is expressed by the microglia and protects photoreceptors in the *rd10* mouse model [6]. The paracrine retinoprotective peptide somatostatin or somatotropin release inhibiting factor (SRIF), is a neuropeptide with a broad inhibitory effect on protein secretion, proliferation, and angiogenesis [20]. The SRIF receptors (SST1-4 in mammals) are distributed in all retinal layers and RPE cells, but the main source of secreted SRIF is the RPE cells [14]. SRIF, via its receptor SSTR2, protects the retina by limiting vascular endothelial growth VEGF, which is normally secreted at the basolateral side of RPE cells, but light-induced damage changes the VEGF secretion to the apical side of RPE cells causing blood-retinal-barrier breakdown and irreversible photoreceptor damage [11]. This indicates that anti-VEGF compounds are of interest therapeutically for retina altered by stress. 

### 2.3. Endocrine Retinoprotective Proteins

Finally, in addition to autocrine and paracrine mechanisms of action, retinoprotective proteins produced and secreted by distal tissues can be transported by the vasculature system to photoreceptors, which characterizes an endocrine signaling function. The deep retinal vessels and the choroidal vessels are the natural delivery ports of retinoprotective proteins from distal tissues to the photoreceptors and the RPE cells [22]. Retinoprotective proteins with endocrine signaling function include those in the proinsulin system, which is one of the firsts to be identified in the retina and protect photoreceptors [23]. All retinal cell layers are insulin-sensitive and express a variety of insulin receptors, however, insulin production within the retina has not been described. Proinsulin and insulin are attenuators of cell death in the developing and adult nervous system [16]. Transgenic expression of human proinsulin in skeletal muscle in the *rd10* mouse model of Retinitis Pigmentosa [17] and in the *P23H* rat model of autosomal dominant Retinitis Pigmentosa [16] delay the death of photoreceptors, prolongs visual function, and attenuates retinal degeneration. Systemic administration of insulin in four mouse models harboring mutations in rod-specific genes (*Pde6b*^−/−^, *Pde6g*^−/−^, *Rho*^−/−^ and *P23H*) prolong photoreceptor survival, whereas depletion of endogenous insulin in these mouse models has the opposite effect [17], exemplifying the importance of the insulin signaling in retinoprotection.

## 3. PEDF

### 3.1. Biogenesis

Among the retinoprotective proteins of interest in ocular research, pigment epithelium-derived factor (PEDF) is relevant due to its well-established protective properties in the retina, in particular the photoreceptors [24,25], RPE cells [26] and in preventing neovascularization of the choroid and retina [27]. A variety of tissues express the gene *Serpinf1* for PEDF, [25] which is altered in retinopathies and in tumor tissues as well [28]. In the eye, the *Serpinf1* gene is transcriptionally active in RPE cells but not detected in the photoreceptors [29]. Both RPE cells and photoreceptors express the gene *Pnpla2* (patatin-like phospholipase domain-containing protein for the PEDF receptor (PEDF-R), a membrane linked enzyme that catalyzes the hydrolysis of phospholipids to release fatty acids and lysophospholipids in retinal cells [30,31].

### 3.2. Structure

**Figure 1:**
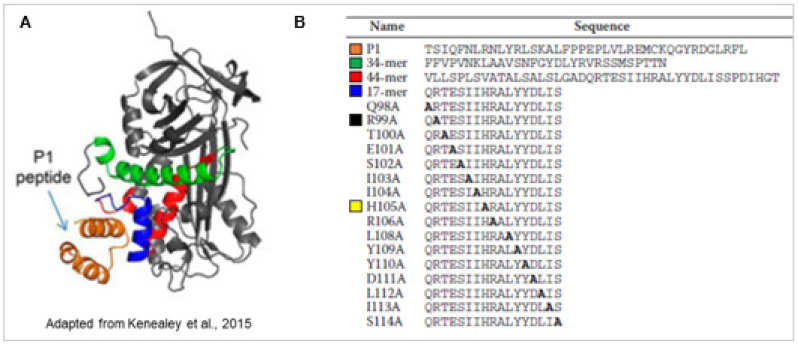
Structure of PEDF and P1 peptide, and sequences of P1 and PEDF-derived peptides. (**A**) Modeling of binding interaction of LBD of human PEDF-R to human PEDF. The structure of the peptide P1 derived from the ab initio fragment assembly protocol of Rosetta is shown in orange. The resultant P1 peptide structure docked to the PEDF crystal structure (Protein Data Bank code 1IMV) using Rosetta program is shown. P1 peptide docked to a cleft that contained a solvent-exposed region corresponding to α-helix C within the residues 98–114 (17-mer; blue) of the neurotrophic 44 amino acid region (red). The antiangiogenic peptide region (34-mer; green) was not part of the docking region. (**B**) Peptides were chemically synthesized and purified. Sequences were from human PEDF-R peptide P1 (Thr210–Leu249) and human PEDF peptides 34-mer (Asp44–Asn77), 44-mer(Val78–Thr121), and 17-mer(Gln98–Ser115) as well as the set of 17-mer alanine scan peptides. The H105A alteration in the 17-mer peptide (yellow) exhibited higher PEDF-R P1 affinity and retinoprotective activity when compared with the unmodified 17-mer, while the R99A alteration (black) abolished the P1 affinity and retinoprotective activity. (Adapted from [32]).

PEDF is a 50 kDa glycoprotein and a member of the serine protease inhibitor (serpin) superfamily. PEDF lacks the serine protease inhibitory activity and is secreted extracellularly, binds PEDF-R with high affinity (Kd = 3 nM) and has cytoprotective activity [30]. PEDF-R is a critical receptor for the survival activity of PEDF. PEDF binding to PEDF-R stimulates the PEDF-R enzymatic activity, and peptides derived from the ligand binding region of the receptor blocks the PEDF·PEDF-R interactions and their mediated retinal survival activities. We have mapped the ligand binding domain (LBD, residue positions T210-L232) in human PEDF-R and termed a blocking peptide derived from this region P1 peptide, T210-L249) [32]. The receptor-binding domain of human PEDF is also identified and termed 17-mer peptide (residue positions Q98–S114). P1 peptide docks to a cleft that contains a solvent-exposed region corresponding to α-helix C within the residues Q98–S114 (17-mer) of the three-dimensional structure of the human PEDF protein [33] (Figure 1). The chemically synthesized 17-mer peptide from the solvent-exposed region of PEDF binds to PEDF-R and exhibits retinoprotective activity [33] (Figure 1). An alanine scanning approach of the 17-mer peptide reveals key interacting residues for PEDF-R binding. The 17-mer peptide with an amino acid alteration at the position 105 from histidine to alanine, 17-mer[H105A], exhibits higher photoreceptor protection efficacy than the unaltered 17-mer, which a blocking peptide P1 and atglistatin, a PEDF-R inhibitor, abolishes. Both the 17-mer and the 17-mer[H105A] peptides protect *rd1* photoreceptors from death in vivo [34]. The recombinant full length PEDF with the H105A amino acids substitution also exhibits affinity for PEDF-R and survival activities to R28 cells and photoreceptors similar and lower than those of their peptide counterparts and unmodified PEDF [33]. 

A comparative study using another serpin, protease nexin-1 (PN-1) shows that its homologous 17-mer region protects R28 retinal cells against serum-starvation-induced cells death in vitro, like full length PN-1, PEDF and the PEDF 17-mer peptides [35]. This observation implies that PEDF serves a model to search for small, pharmacologic peptide candidates in other proteins of the serpin family, such as PN-1. Given that the serpin PN-1 inhibits serine protease thrombin and that the region to inhibit this protease is not present in the 17-mer, it indicates that inhibition of proteases by PN-1 is dispensable for cytoprotective activity. In addition, the PN-1 variant that lacks inhibitory activity due to an alteration R346A in the inhibitory site does not lack cytoprotective activity. This further implies that PN-1 mechanism of cytoprotection is independent of serine protease inhibition like PEDF [35]. 

Important interactions of PEDF with the extracellular matrix (ECM) components such as collagens occur [36]. The crystal structure of PEDF in a complex with a disulfide cross-linked heterotrimeric type I collagen peptide shows that PEDF specifically interacts with an amphiphilic sequence of the type I collagen in the ECM. This indicates that collagen I in the ECM functions as an extracellular reservoir of PEDF that is slowly and temporally released by the ECM remodeling such as collagen turnover by metalloproteinases [37].

### 3.3. Mechanisms of Action

PEDF and the PEDF peptides 17-mer and 17-mer[H105A] protect photoreceptors from damage in the *rd1* and *rd10* mouse models of retinal degeneration [29,34], in the *Mitf* knockout mouse model of microphtalmia [38], and a focal light-emitting diode (LED)-induced phototoxicity mouse model [39]. These effects are mediated by binding of the extracellular PEDF to the cell surface PEDF-R and promoting intracellular signaling cascade that ends in photoreceptor cell survival. The direct intracellular targets of PEDF-R upon PEDF binding are beginning to be elucidated. The PEDF/PEDF-R axis protects photoreceptors of the *rd1* mouse model by reducing intracellular calcium in the degenerating retina and engaging calpains, BAX and AIF proteins [40]. Other pathways described using models of retinoprotection in vitro have been reviewed elsewhere [25] and include PI3K/AKT, ERK1/2 and the docosahexaenoic acid (DHA)/neuroprotectin D1 (ND-1) signaling pathways that are also downstream effectors of the PEDF/PEDF-R complex.

The generation of the *Serpinf1* knockout mouse is useful to address mechanisms of action for PEDF. The *Serpinf1* knockout in a *rd10* mouse background shows that the absence of PEDF affects the magnitude of photoreceptor degeneration [29]. These findings highlight the importance of PEDF as a natural signaling protein in the retina to mitigate an undergoing degenerative process in vivo.

Besides the retina, it is worth mentioning the neurotrophic properties of PEDF in other tissues. PEDF combined with DHA promotes corneal nerve regeneration and upregulates the expression of the retinoprotective genes *Vip*, *Bdnf* and *Ngf* (retinoprotective proteins discussed above) and axon growth protein semaphorin 7a (*Sema7a*) during the corneal nerve regeneration [41]. Administration of the 44-mer PEDF peptide to the cornea of rabbits also induces the corneal nerve regeneration following injury created by corneal epithelial debridement [42]. 

### 3.4. Regulation of PEDF Expression

In murine eyes with retinal degeneration, PEDF levels are lower than in their wild type counterparts [34,43], corroborating the fact that PEDF levels are also decreased in eyes affected by retinal degenerative processes in humans [43,44,45,46]. The levels of PEDF in eyes of patients with retinitis pigmentosa are significantly lower than those in eyes without this disease [46]. The vitreous of patients with choroidal neovascularization due to age-related macular degeneration contains lower PEDF levels and lacks the antiangiogenic activity of vitreous from age-matched controls [44]. These observations suggest that loss of PEDF creates a permissive environment for retinal degeneration and ocular neovascularization.

Engineering PEDF-expressing RPE cells for cell therapies can enhance PEDF production in the retina. The apical secretion of PEDF by RPE cells [47] can serve to inhibit invasive choroidal neovascularization into the posterior retina and in turn maintain its avascularity and structural integrity. PEDF production and apical secretion by RPE cells are also important phenotypic markers for the differentiated and polarized status of RPE cells in vitro, as shown with ARPE-19 cells [48] and RPE cells derived from human pluripotent stem cells [49]. 

Furthermore, a recent study using a rat NaIO_3_-induced retinal degeneration model provides evidence of epigenetic regulatory mechanisms based on micro-RNA biology for PEDF expression in the RPE cells. The expression of the micro-RNA miR-25, which contains a seeding region that directly binds to PEDF mRNA and prevents its translation, is upregulated after induction of retinal degeneration [50]. PEDF mRNA is also a direct target for the micro-RNA miR-320c [51]. This micro-RNA is upregulated in nasopharyngeal carcinoma cells and is involved in decreasing PEDF expression and epithelial-to-mesenchymal transition that contributes to tumor metastasis [51]. This new regulatory level by miR-320c needs to be confirmed in the retina and exemplifies the increasing relevance of epigenetic events in regulating PEDF expression.

## 4. PEDF Delivery Systems

**Figure 2:**
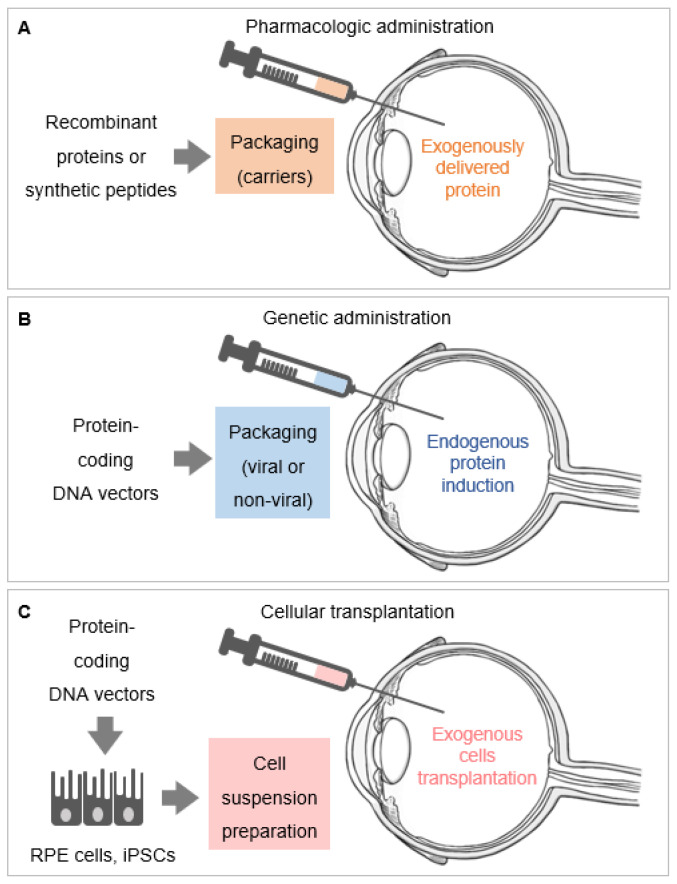
Systems of local delivery of retinoprotective proteins. (**A**) Pharmacologic administration of retinoprotective proteins, which consists of delivering purified recombinant proteins and synthetic peptides. These can be packaged into carriers (nanoparticles, microparticles, microemulsions) before administration and delivered into the vitreous (as exemplified in the figure). (**B**) Genetic administration of retinoprotective proteins is accomplished by using DNA expression vectors packaged into viral and non-viral carriers to induce the endogenous expression of these proteins. (**C**) The genetic administration of retinoprotective proteins can be performed in exogenous cells in vitro, via DNA expression vectors packaged into viral and non-viral carriers, in order to induce the production of recombinant retinoprotective proteins. These exogenously transfected or infected cells, exemplified by retinal pigment epithelium (RPE) and induced pluripotent stem cells (iPSCs), are then transplanted into the retina. Only one route of delivery (intravitreal injection) is shown in the Figure 2A–C; other routes of delivery of retinoprotective proteins include injections in the subretinal space or in the subconjunctiva, and the use of topical corneal drops.

We focus on PEDF delivery systems reported during the last five years. Figure 2 summarizes three systems currently used to deliver PEDF in the retina in vivo.

### 4.1. Pharmacological Administration of PEDF

PEDF is obtained as a recombinant protein purified from bacterial and mammalian cells. Its derived peptides are chemically synthesized and purified. The routes of drug administration in vivo can be systemic or local such as suprachoroidal, intravitreal, subtenon or subretinal injections, and ectopic (corneal) application and have been extensively reviewed elsewhere [52]. The ectopic delivery of PEDF protein and peptides is performed in vivo with or without the assistance of molecular carriers (Table 1). Carriers can increase the stability of PEDF, which has a relatively short physiologic half-life [53] and prevent the need of repeated injections of PEDF. Table 2 shows a list of recent reports in which pharmacological administration of recombinant PEDF and its corresponding peptides was performed in vivo. 

The Table 2 describes experimental models, molecules (PEDF and/or corresponding peptides), types of carrier (when used), routes of PEDF delivery and effect on target cells. Intravitreal injections of PEDF without the use of a carrier remains the most common approach for the pharmacologic administration of PEDF in vivo [34,38,40,54]. Ectopic (eye drops) administration of PEDF has also been used [38,52]. Photoreceptors, and endothelial cells (highlighted in green, and in pink, respectively) are the main targets of pharmacologic administration of PEDF in vivo. Retinal degeneration models such as *rd1* [40] and *rd10* mice [34] are useful to provide mechanistic insights into PEDF retinoprotection. Oxidative stress injury mouse models are used to investigate the effects of PEDF in preserving photoreceptors integrity [54]. Carriers such as polymer-conjugated ester prodrug [55] and Type I collagen [56] are used in rabbit and in mouse, respectively, to examine the inhibitory effects of PEDF against choroidal neovascularization.

### 4.2. Genetic Administration of PEDF and Cell-Based Therapies

The administration of genes for production of retinoprotective proteins provide a sustained endogenous expression and therefore avoid the need of multiple administrations. There are challenges associated with the genetic administration of vectors such as targeting specific cells in the retina, e.g., photoreceptors or RPE cells, and safety due to cellular toxicity associated with viral vectors. Table 3 summarizes studies of genetic administration of PEDF in vivo reported during the last five years. It includes organism model, DNA vectors, carriers/host cells, routes of delivery, and effect (including target cells) for genetic administration of PEDF in vivo. 

Cell-based delivery systems are also used to administer PEDF to the retina, by first infecting placental-derived [60], human mesenchymal-derived (hMSC) [59] and neural [58] stem cells with viral vectors, or transfecting primary rat RPE cells [62] in vitro with expression vectors, and generating in vitro PEDF-secreting cells that can be transplanted in the vitreous or in the subretinal space in vivo. The cell-based delivery systems of PEDF reported during the last five years are summarized in the Table 3. Most of these studies employ viral vectors to pack PEDF-coding DNA [58,59,61,63], and in vivo infections using mouse and rat models of retinal degeneration and neovascularization. Approaches using cell-based genetic administration of PEDF from mesenchymal stem cells, and subsequently implanting these PEDF-producing cells in the subretinal space or in the vitreous are emerging in studies aiming at the regeneration of the RPE layer. Non-viral carriers for cellular transfections such as AMAXA Nucleofector technology [60] are alternatives to viral infections to transfect PEDF expression vectors. Different types of PEDF-coding, non-viral vectors such as the non-integrating episomal vector pEPito [57] and the non-viral, hyper-active sleeping beauty transposon [62] are used to engineer PEDF-expressing cells for potential cell-based therapies.

## 5. Future Directions for Protein Delivery Systems in Ocular Research

The research on pharmacologic and genetic administration of retinoprotective proteins has had unprecedented success in generating different treatments toward the recovery of vision loss. Regarding the pharmacologic approach using exogenously prepared proteins and peptides as cargo molecules, a variety of neuron-targeted nanoparticles are available to deliver the cargo and to enhance neuronal protection [64]. Significant progress includes the understanding of the uptake mechanism and the physicochemical properties of nanoparticles and microparticles to encapsulate proteins [64]. The use of novel delivery methodologies, such as microneedles and intravitreal implants that allow the gradual intraocular release of proteins and carriers that are specifically designed for ocular research [65] are increasing the efficacy of the pharmacologic administration of retinoprotective proteins to target retinopathies. 

Encapsulation of proteins with nano-formulation shows efficient delivery of retinoprotective proteins. For example, the intravitreal delivery of the neuroprotective peptide erythropoietin (EPO) is improved with the use of microparticles formulated with EPO and the hydrolytically degradable poly(lactic-co-glycolic acid) (PLGA) or the reactive oxygen species (ROS)-degradable poly(propylene sulfide) (PPS) in models of blast injury-induced retinal damage [66]. This research warrants the use of encapsulating PEDF in similar microparticles system to control and extend its release locally using in vivo retinopathy mouse models. 

In addition to encapsulating retinoprotective proteins with nano-formulated microparticles, microemulsions (ME) [67,68] and hydrogels [68,69] are also available and known to behave as “smart carriers”. They change phase upon physiological conditions (water content, temperature) and undergo phase-transition from a water-in-oil state to a liquid crystalline state and then to coarse emulsion with different viscosities. This allows slower release of cargo molecules in the eye, which include druggable compounds and retinoprotective proteins. The various sizes of ME, from 5 to 200 nm [67], and their long-term stability make ME and hydrogels compelling delivery system employing the full length PEDF or its derived bioactive peptides for future research.

Regarding the genetic administration of PEDF, viral and non-viral vectors are successfully employed to introduce PEDF expression in retinal and RPE cells (Table 2). As precedent, an adeno-associated virus (AAV-2)-based gene therapy, Vortigern Neparvovec (Luxturnatm), is already approved by the FDA for the treatment of Leber Congenital Amaurosis (LCA) [70]. Despite the advantages of the AAV systems, such as the induction of sustained gene expression and recombinant protein production of a protein, there are safety concerns with toxicity and mutagenesis due to the integration of the virus into the genome, which could cause long term, unpredictable effects. Another limitation for the use of this AAV system is the maximal size of the DNA coding construct (approximately 5kB) that can be packaged into an AAV [71]. There is an increasing need to develop genetic delivery systems that overcome these limitations.

Moreover, it is possible to modify the local expression of naturally occurring proteins by editing their genes. The use of CRISPR-based editing approaches [72] and innovations in designing expression vectors containing both *Cas9* and small guiding RNAs (sgRNAs), are now commonly used to infect retinas in vivo in order to edit mutated rhodopsin [73]. CRISPR-based approaches could be employed to edit the endogenous PEDF expressed in RPE cells in vivo, enhancing PEDF activity or increasing its production. Second generation PEDF with alterations that increase its neurotrophic activity are excellent choices for this approach, such as the single amino acid alteration in PEDF[H105A] [33]. Alterations of amino acids to increase the stability of PEDF without affecting its biological activity are also considered of interest for this approach.

Finally, methods of mRNA encapsulation in nanoparticles currently applied in mRNA vaccine technologies against several types of infectious diseases and cancer [74] offer an attractive alternative to viral systems for the genetic administration of retinoprotective genes in ocular research. Such mRNA-based approaches involve relatively novel concepts of RNA-based therapies that include methods in mRNA design and formulation of nanoparticles to encapsulate the mRNA of interest. An example of this methodology that could potentially be applied to the genetic administration of PEDF is the use of the cationic peptide protamine to transfect PEDF-encoding mRNA constructs in vivo [75].

## 6. Concluding Remarks

The use of retinoprotective proteins in ocular research is currently benefiting from the advances in research on specific carriers that can significantly increase the delivery of these proteins in the vitreous or in the retinal and RPE layers of the eye. Challenges associated with the release and the extracellular stability of these proteins are being addressed by designing packaging carriers that protect the retinoprotective proteins from proteolysis, and the development of slow release carriers that avoid the need of multiple injections of administration for a sustained release of these proteins. The advantage of pharmacologic delivery of retinoprotective proteins when compared with gene and cell therapies, includes the safety of delivering a purified drug without altering the genetic component of the endogenous cells, and/or avoiding rejection as a possible transplantation side effect, respectively. 

Among retinoprotective proteins, PEDF is attractive for in vivo studies using pharmacologic or genetic delivery systems and cell therapy strategies. There are several advantages for the use of PEDF delivery systems: 1. PEDF is a retinoprotective protein that naturally occurs in the eye and is present in the aqueous humor of humans [76]; 2. PEDF is nontoxic [77]; 3. PEDF is multimodal and possesses neurotrophic [27], antiangiogenic [55], anti-inflammatory [78], antioxidant properties [79,80], all of which are beneficial for the treatment of several multifactorial retinal diseases; 4. PEDF is a soluble protein that can diffuse from the vitreous to the back of the eye and from the subconjuntiva to the retina [81,82,83,84]; 5. The modular PEDF can be fragmented to yield peptides with distinct individual activities [33]. However, some disadvantages are also associated with PEDF, such as: 1. Some of the mechanisms of action of PEDF remain to be elucidated; 2. PEDF clearance in the eye can occur within the first 24 h of administration and may require sustained release upon pharmacological delivery [82]; 3. Due to its multimodal properties [33], the use of PEDF as a full length protein could trigger undesirable side effects, which warrants the use of its relevant smaller peptides for an individual activity.

## Figures and Tables

**Table 1:** Representative examples of mechanisms of action for retinoprotective proteins *.

Mechanism of Action	Retinoprotective Protein	Abbreviation	Expressing	Target	Ref.
			Cells	Cells	
Autocrine	insulin-like growth factor 1	IGF-1	photoreceptors	photoreceptors	[6]
	fibroblast growth factor 2	FGF-2	photoreceptors	photoreceptors	[7]
	ciliary neurotrophic factor	CNTF	photoreceptors	photoreceptors	[8]
	brain-derived neurotrophic factor	BDNF	photoreceptors	photoreceptors	[9]
	nerve Growth factor	NGF	photoreceptors	photoreceptors	[10]
	vascular endothelial growth factor	VEGF	photoreceptors	photoreceptors	[11]
Paracrine	pituitary adenylate cyclase-activating polypeptide	PACAP	retinal cells	photoreceptors	[12]
	vasoactive intestinal peptide	VIP	(except photoreceptors)	photoreceptors	[13]
	insulin-like growth factor 1	IGF-1	amacrine cells	photoreceptors	[6]
	somatostatin, somatotropin release inhibiting factor	SRIF	microglia	photoreceptors	[14]
	vascular endothelial growth factor	VEGF	RPE	choroidal endothelium	[15]
Endocrine	proinsulin	pro-INS	skeletal muscle	photoreceptors	[16,17]
	insulin	INS	none (systemic administration)	photoreceptors	[17]

* Color-code shows the mechanism of action: autocrine (green), paracrine (purple) and endocrine (pink).

**Table 2:** Pharmacologic administration of PEDF and derived peptides in vivo *.

Organism Model	Molecules	Carrier	Route of	Effect	Ref.
			Delivery		
mouse LED-induced	rhuPEDF	none	intravitreal	photoreceptor survival	[39]
Phototoxicity	17-mer 17-mer[H105A]		injection		
mouse *Mitf* KO-induced microphtalmia	rhuPEDF	none	eye drops	photoreceptor survival	[38]
	17-mer				

mouse retinal	rhuPEDF44-mer	none	intravitreal	photoreceptor survival	[34]
degeneration (*rd10*)	17-mer		injection		
	17-mer[H105A]				
mouse retinal degeneration (*rd1*)	rhuPEDF	none	intravitreal	photoreceptor survival	[40]
	17-mer		injection		
	17-mer[H105A]				
mouse oxidative stress	rhuPEDF	none	intravitreal	photoreceptor survival	[54]
injury (H_2_O_2_)			injection		
rabbit laser-induced	8-mer (peptide 335, modified from 34-mer)	polymeric	intravitreal	choroidal neovascularization inhibition	[55]
choroidal		ester	injection		
neovascularization		prodrug			
mouse laser-induced	34-mer	type I	intravitreal	choroidal neovascularization inhibition	[56]
choroidal		collagen	injection		
neovascularization			eye drops		

* The color code indicates the target cells for the pharmacologic administration of PEDF in vivo (green: retinal cells; pink: endothelial cells); rhu: recombinant, human.

**Table 3:** Genetic administration of PEDF in vivo *.

Organism Model	DNA Vector	Carrier/Host Cells	Route of Delivery	Effect	Ref.
*Ins2*^Akita^ diabetic mouse	pEPito-hCMV-PEDF episomal vector	none	subretinal injection	photoreceptors survival	[57]
		(electroporation)			
rat optic nerve crush	PEDF-secreting neural stem cell (NSC)-based system, lentivirus	none	subretinal cell transplantation	photoreceptors survival	[58]
		host cells: human neural stem cells			
rat optic nerve crush	AAV2.PEDF combined with human mesenchymal stem cell	none	Intravitreal cell transplantation	RGC survival and ON injury	[59]
		host cells: human mesenchymal stem cells			
rat H_2_O_2_-induced retinal degeneration	Placenta-derived-mesenchymal stem cells overexpressing PEDF plasmid	none	Intravitreal cell transplantation	Mitochondrial biogenesis in RPE cells	[60]
		host cells: human placental-derived stem cells			
mouse laser-induced neovascularization	multigenic AAV5.PEDF + multiple miRNAs targeting the VEGF-A gene, p/miR(5,B,7/Irr)-AsR/PEDF-PE	none	Intravitreal injection	Choroidal cells inhibition	[61]
rat choroidal neovascularization	IPE or RPE cells with pFAR4-ITRs CMV PEDF BGH plasmid, sleeping beauty transposon	none	Subretinal cell transplantation	choroidal cells inhibition	[62]
		(electroporation)			
		host cells: primary rat RPE cells			
rat choroidal neovascularization	Lentivirus-*PEDF*-green fluorescent protein (GFP)	none	Intravitreal injection	choroidal cells inhibition	[63]

* The color code indicates the target cells for these studies (green: retinal photoreceptor cells; purple: RPE cells; pink: endothelial cells). When cell therapies are associated with the genetic delivery of PEDF, the host cells to be transplanted are indicated.

## Data Availability

Not Applicable.
